# Ion-Imprinted Polymer-on-a-Sensor for Copper Detection

**DOI:** 10.3390/bios12020091

**Published:** 2022-02-02

**Authors:** Zeynep Gerdan, Yeşeren Saylan, Mukden Uğur, Adil Denizli

**Affiliations:** 1 Graduate School of Biomedical Engineering, Istanbul University-Cerrahpaşa, Istanbul 34320, Turkey; zeynep.gerdan@ogr.iu.edu.tr; 2 Department of Chemistry, Hacettepe University, Ankara 06800, Turkey; yeseren@hacettepe.edu.tr; 3 Department of Robotics and Intelligent Systems, Institute of Science, Turkish German University, Istanbul 34820, Turkey; mugur@tau.edu.tr

**Keywords:** copper detection, ion detection, ion-imprinted polymer, plasmonic sensor

## Abstract

The accumulation of metal ions in the body is caused by human activities and industrial uses. Among these metal ions, copper is the third most abundant ion found in the human body and is indispensable for health because it works as a catalyst in the iron absorption processes. However, high doses of copper ions have been reported to generate various diseases. Different types of sensors are used to detect metal ions for several applications. To design selective and specific recognition sites on the sensor surfaces, molecular imprinting is one of the most used alteration methods to detect targets by mimicking natural recognition molecules. In this study, an ion-imprinted polymer-integrated plasmonic sensor was prepared to selectively detect copper (Cu(II)) ions in real-time. Following different characterization experiments, the Cu(II)-imprinted plasmonic sensor was employed for kinetic, selectivity, and reusability studies. According to the results, it was observed that this sensor can measure with 96% accuracy in the Cu(II) concentration range of 0.04–5 μM in buffer solution. The limit of detection and limit of quantification values were computed as 0.027 µM and 0.089 µM. The results also showed that this plasmonic sensor works successfully not only in a buffer solution but also in complex media such as plasma and urine.

## 1. Introduction

Copper is one of the heavy metal ions that have a critical role in many environmental and medical applications [1,2]. It causes environmental chemical pollution, and high concentrations of copper are dangerous for living organisms because of the accumulation inside of the organism, which gives rise to a severe form of poisoning [3,4]. Copper also acts a necessary role in several physiological processes such as cellular respiration, bone formation, and connective tissue development. However, in the presence of high doses of copper in the human body, health is adversely affected [5,6]. It also causes gastrointestinal diseases, liver damage, and serious neurodegenerative diseases [7,8]. Along with the common applications of copper in agriculture and industry, the possible toxic impacts on humans have drawn increasing attention. Thus, preparing practical and simple devices for real-time detection of copper is vital for both human health and environmental pollution. For the detection of copper, there are several techniques, including inductively coupled plasma mass spectrometry, atomic absorption/emission spectroscopy, colorimetric assays, and sensors [9,10,11,12].

Plasmonic-based sensors are sub-classes of optic sensors. Surface plasmon resonance is designed to measure changes in the refractive index of surface plasmon on a metal surface [13,14]. Moreover, surface plasmon resonance characterizes the interactions between analytes immobilized on the metal and the receptor, and it has unique advantages such as real-time and rapid measurement, high sensitivity and stability, and no need for specific labeling [15,16,17]. One of the most common surface modification methods, molecular imprinting, is mainly based on molecular and specific recognition of the target molecule [18,19,20,21]. This method is widely used owing to its remarkable properties such as being fast, user-friendly, and selective. Due to its chemical and physical durability, low cost, high stability, and reusability features, molecularly imprinted polymer-on-a-sensor have become very interesting modalities for different utilizations [22,23,24,25,26,27,28].

In this study, we designed an ion-imprinted polymer integrated plasmonic sensor to detect copper (Cu(II)) ions. After the preparation and characterization steps, the Cu(II)-imprinted plasmonic sensor was used for adsorption and kinetic studies. Different concentrations of Cu(II) samples were employed in the plasmonic sensor to investigate detection performance and compute binding kinetics parameters. The Cu(II)-imprinted plasmonic sensor was also validated with artificial plasma and urine samples.

## 2. Materials and Methods

### 2.1. Materials

Copper nitrate hemipentahydrate (Cu(NO_3_)_2_·2.5H_2_O), zinc nitrate (Zn(NO_3_)_2_), nickel nitrate hexahydrate (Ni(NO_3_)_2_·6H_2_O), ferric nitrate (Fe(NO_3_)_3_·9H_2_O), lithium nitrate (LiNO_3_), cadmium nitrate tetrahydrate (Cd(NO_3_)_2_·4H_2_O), lead-(II)-nitrate (Pb(NO_3_)_2_), ethylenediaminetetraacetic acid (EDTA, C_10_H_16_N_2_O_8_), 2-hydroxy ethyl methacrylate (HEMA, C_6_H_10_O_3_), ethylene glycol dimethacrylate (EGDMA, C_10_H_14_O_4_), 2,2′-azobis (2-methylpropionitrile) (AIBN, C_8_H_12_N_4_), 2-propene-1-thiol (allyl mercaptan, C_3_H_6_S), dipotassium hydrogen phosphate (K_2_HPO_4_) and potassium dihydrogen phosphate (KH_2_PO_4_) were obtained from Sigma-Aldrich. Ethanol (C_2_H_5_OH), hydrogen peroxide (H_2_O_2_), sodium chloride (NaCl), sodium hydroxide (NaOH), sulfuric acid (H_2_SO_4_), and acetic acid (CH_3_COOH) were obtained from Merck and Fluka.

### 2.2. Modification and Preparation of Plasmonic Sensors

The modification of the commercial gold surface was performed with allyl mercaptan by dropping it onto the surface of the plasmonic sensor and incubating overnight. As a result of the modification, unsaturated bonds were formed by attaching allyl groups for polymerization to take place on the gold surface. Conjugated polymers such as polythiophene, polyaniline, and polypyrrole are commonly employed to design sensor surfaces [29]. However, there are some modification challenges to be considered [30]. Herein, an amino acid-based functional monomer (*N*-methacryloyl-L-cysteine methyl ester, MAC) was used for polymerization. MAC is a cysteine derivative and metal complexing functional monomer which used for a metal-complexing ligand preparation in the pre-complex step with high selectivity for Cu(II) ions. For this aim, it was first synthesized following the procedure [31], and the optimum MAC-Cu(II) pre-complex was evaluated by ultraviolet-visible spectroscopy.

Following the addition of co-monomer (HEMA) and cross-linker (EGDMA), the pre-complex was mixed with monomer solution. HEMA is a vinyl monomer that can be easily polymerized and poly(HEMA) has hydrophilic character, minimal non-specific interactions, nontoxic, biocompatible, high chemical and mechanical stability, and resistance toward environmental attacks. EGDMA is a diester formed by condensation of two equivalents of methacrylic acid and one equivalent of ethylene glycol. It makes the polymer chain bind to another and determines the features of imprinted polymers to obtain the structural integrity of the imprinted binding sites [32].

**Figure 1:**
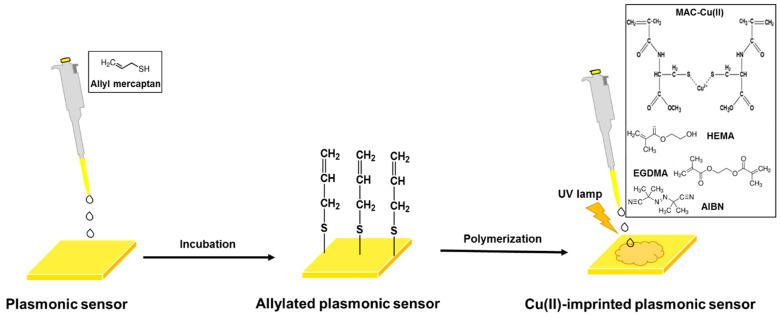
Schematic representation of plasmonic sensor preparation.

Then the initiator (AIBN) was added to the monomer solution, and 10 μL of the mixture was dropped onto the allylated plasmonic sensor surface. The bulk polymerization was completed under ultraviolet light (Figure 1). The unreacted monomer was washed using ethyl alcohol. The Cu(II)-imprinted plasmonic sensor was interacted with a desorption solution (0.01 M EDTA) to remove the Cu(II) ions. A non-imprinted plasmonic sensor was also developed with the same process without using Cu(II) ions.

### 2.3. Characterization of Plasmonic Sensors

Characterizations of bare, allylated, non-imprinted, and Cu(II)-imprinted plasmonic sensors were performed via atomic force microscope (AFM), ellipsometry, contact angle analysis, and attenuated total reflection-Fourier transform infrared (ATR-FTIR) spectroscopy, respectively. The morphology of polymers on the sensor surfaces was investigated by using tapping mode for AFM analysis in two and three dimensions with high resolution. The polymer thickness of sensor surfaces was determined using an ellipsometer with an auto-nulling imaging property. Contact angle analyses were carried out employing a sessile drop to determine the surface features of plasmonic sensors. The ATR-FTIR spectra were obtained in the wavenumber range of 400–4000 cm^−1^ on the plasmonic sensor surfaces, and the total amount of reflection on the surfaces was measured.

### 2.4. Kinetic Analysis of Plasmonic Sensors

Following the characterization studies of plasmonic sensors, the kinetic analysis was performed by using the SPRimager II instrument (GWC Technologies, Madison, WI, USA). The gold plasmonic sensor surfaces (SPR-1000-050), with dimensions of 1 × 18 × 18 mm^3^ and a thickness of 50 nm gold, and an SF10 equilateral prism were also provided by the GWC Technologies. During the image acquisition, the changes in reflectivity (ΔR) values of the light were recorded by changing the angle of incidence of the light to the surface of the Cu(II)-imprinted plasmonic sensor (48.7–56.7°). Thus, the surface plasmon curves, its slope, and the number of changes in reflectivity against the angle of incidence of light were reported by plotting. In this SPRimager II instrument, the primary plasmonic response is in pixel intensity unit (PIU). The signal changes obtained in PIU were converted to the real change in reflectivity, ΔR.

The plasmonic sensors were equilibrated with water and buffer solution in the same flow rate (500 μL/min) for each experiment. While buffer solution was passed through the sensor system, the surface plasmon curves were obtained, and the resonance refraction angle was determined. All kinetic analyses were carried out at this angle. The buffer solution passed through the system using a pump for 200 seconds (s) for obtaining a baseline curve. The ΔR were achieved by the plateau values in 300 s for each study, and then desorption solution (0.01 M EDTA) was used for 150 s.

Kinetic analyses were applied using the same Cu(II) solution (4 µM) for different buffer solutions (4.0–8.0) to find the highest response of the Cu(II)-imprinted plasmonic sensor. Then, different Cu(II) solutions (0.04–5 µM) were used in a buffer solution with a pH of 8.0 for obtaining the calibration curve of a Cu(II)-imprinted plasmonic sensor.

Selectivity analysis of non-imprinted and Cu(II)-imprinted plasmonic sensors was performed separately using competitive ions (Fe(II), Cd(II), Li(I), Ni(II), and Pb(II)) solutions, which were prepared with the same concentration (5 µM) in a pH 8.0 buffer solution. The selectivity (k) and relative selectivity (k’) coefficients were calculated. The selectivity coefficient (k), which expresses the interaction of the Cu(II) ion with the Cu(II)-imprinted plasmonic sensor in the presence of competing agents, is calculated using Equation (1).

k = k_Cu(II) ion_/k_competitive ion_ (1)

With the Cu(II)-imprinted plasmonic sensor, the k-values of other ions are used to determine whether the quenching selectivity is high. The relative selectivity coefficient (k′) is calculated using Equation (2).

k′= k_Imprinted_/k_Non-imprinted_ (2)

Reusability performance of the Cu(II)-imprinted plasmonic sensor was obtained with the same (4 µM) and different (3–5 µM) concentrations of Cu(II) solutions, respectively. Moreover, the storage stability performance of the Cu(II)-imprinted plasmonic sensor was also examined with the same Cu(II) concentration (4 µM) at different times (0–36 months). Finally, the artificial plasma and urine samples were prepared by adding 10 µL of each sample to the 5 µM concentration of Cu(II) in pH 8.0 buffer solution to understand the working performance of the Cu(II)-imprinted plasmonic sensor in complex samples.

## 3. Result and Discussions

### 3.1. Characterization of Plasmonic Sensors

The pre-complex samples were prepared with a functional monomer, MAC and template molecule, Cu(II) using different ratios (0.5:1.0, 1.0:1.0, 2.0:1.0 and 3.0:1.0). Then, an optimum pre-complex ratio was chosen as 2.0:1.0, which has 0.1 mmol MAC and 0.05 mmol Cu(II). The increase of absorbance intensity ended up at this ratio, and polymerization was carried out using this ratio (Figure S1).

The functional group investigation of the MAC monomer, Cu(II)-imprinted and non-imprinted plasmonic sensors were performed using ATR-FTIR spectroscopy (Figure S2). The ATR-FTIR spectrum of MAC had characteristic stretching vibration carboxyl-carbonyl and amide bands at 1738 and 1521 cm^−1^, respectively. The S-H bending peak appeared at 2982 cm^−1^ of MAC. The characteristic carboxyl-carbonyl and amide bands of Cu(II)-imprinted plasmonic sensors were observed at 1716 and 1530 cm^−1^, respectively. The shifting bands from 1738 to 1716 cm^−1^ and from 1521 to 1530 cm^−1^ confirmed that the imprinting process succeeded. The broad -OH group band appears in the same region as the stretching band of the -NH group at 3414 cm^−1^. The non-imprinted plasmonic sensor surface, which has no bond with Cu(II), the band at 1721 and 1577 cm^−1^, formed a peak for the carboxyl-carbonyl and amide bands, which was formed by the incorporation of the MAC monomer into the HEMA monomer. Thus, specific bands of polymers on Cu(II)-imprinted and non-imprinted plasmonic sensors showed that polymerization took place successfully.

**Figure 2:**
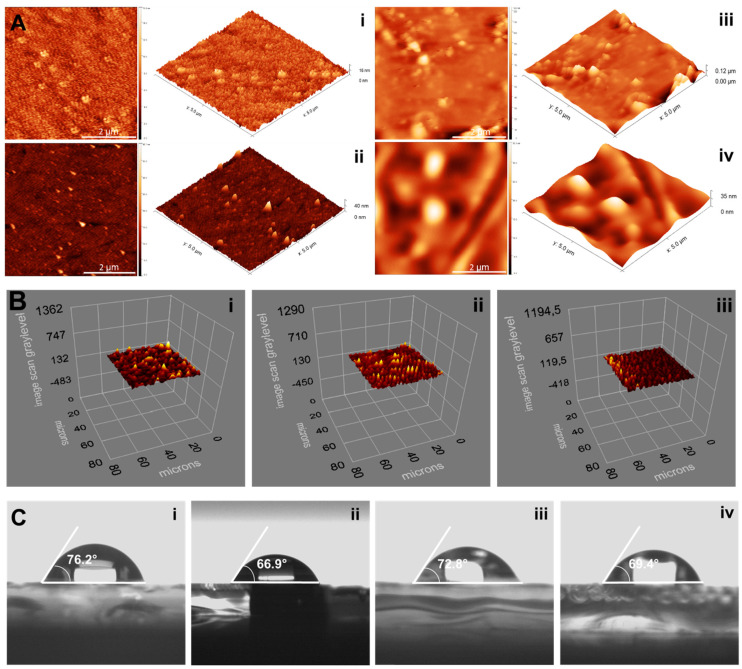
AFM (**A**: bare (**i**), allylated (**ii**), Cu(II)-imprinted (**iii**) and non-imprinted (**iv**)) ellipsometry (**B**: allylated (**i**), Cu(II)-imprinted (**ii**) and non-imprinted (**iii**)) and contact angle (**C**: bare (**i**), allylated (**ii**), Cu(II)-imprinted (**iii**) and non-imprinted (**iv**)) images of plasmonic sensors.

The morphological properties of bare, allylated, Cu(II)-imprinted and non-imprinted plasmonic sensor surfaces were investigated by AFM analysis. The mean roughness values were measured as 1.4 ± 0.2 nm, 2.2 ± 1.1 nm, 5.8 ± 2.3 nm, and 5.1 ± 1.9 nm, respectively. These results showed that polymers were successfully and homogeneously synthesized on the gold surfaces of plasmonic sensors (Figure 2A).

The thickness values of allylated, Cu(II)-imprinted and non-imprinted plasmonic sensor surfaces were determined as 75.1 ± 2.7 nm, 87.6 ± 2.6 nm, and 82.5 ± 2.9 nm by ellipsometry analysis (Figure 2B). The increase in the thickness values indicated that the polymerization was also accomplished and is compatible with the AFM results.

The contact angle values of bare and allylated plasmonic sensor surfaces were obtained as 76.2° ± 0.3 and 66.9° ± 1.2, respectively. The decrease in the value of the contact angle means that the hydrophilic property of the surface increases. The presence of thiol groups in the chemical structure of allyl mercaptan used in the surface modification process caused a decrease in the contact angle value. Moreover, as depicted in Figure 2C, the contact angle values of Cu(II)-imprinted and non-imprinted plasmonic sensor surfaces were evaluated as 72.8° ± 2.3 and 69.4° ± 2.1, respectively. The functional monomer, MAC, used for the construction of Cu(II)-imprinted and non-imprinted plasmonic sensors is a cysteine-based monomer and highly hydrophilic. For this reason, the bonding of a hydrophilic polymer to the surface created an increase in the hydrophilic property of the surface and decreased the contact angle.

### 3.2. Kinetic Analysis of Plasmonic Sensors

Plasmonic sensors are employed for the detection of several molecules and ions. To measure the kinetic response of a Cu(II)-imprinted plasmonic sensor, the binding of Cu(II) ions was monitored using a plasmon system. For this aim, different pHs (4.0–8.0) were used to obtain a maximum response. As depicted in Figure S3, the highest signal response (ΔR) was obtained in a pH 8.0 phosphate buffer. The choice of complexing monomer is derived from the high affinity of side-chain sulfhydryl groups in the MAC towards Cu(II) ions. In a typical ion-imprinting process, the MAC was complexed with the Cu(II) ion before the polymerization process to form a non-covalent coordination complex. MAC has cysteine, and cysteine contains a sulfhydryl group, which is primarily responsible for interaction with metal ions. This group could serve as a coordination site for metal chelation. Since the pK_a_ of the thiol group, which is the R group of cysteine, is 8.33, Cu(II) and thiols made a strong bilateral coordination in the pH 8.0 phosphate buffer. In addition, since the pH of 8.0 is above the isoelectric point of cysteine (pI 5.02), cysteine will be negatively charged, thus allowing easy bonding with the positively charged Cu(II) ion [33,34].

**Figure 3:**
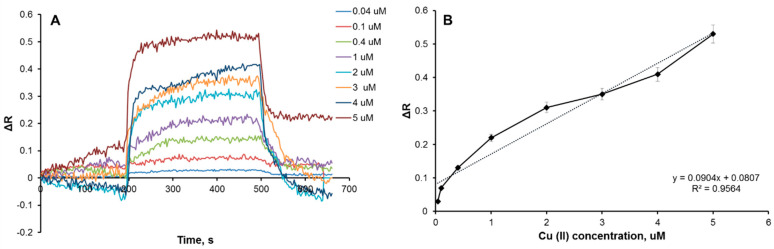
Real-time Cu(II) detection (**A**) and calibration curve (**B**) of Cu(II)-imprinted plasmonic sensor.

An increasing concentration of the Cu(II) ion causes to ΔR increase. The change in concentration is a driving force for the signal. The response rises in parallel with the concentration rise because of the differences of concentration between solid and liquid phases of the Cu(II) ions through the sensor surface [35]. The plasmonic sensor showed linearity in the range of 0.04–5 µM (Figure 3A). The equation of calibration curve in this range was determined as y = 0.0904x + 0.0807, and its correlation coefficient (R^2^) was computed as 0.96 (Figure 3B), which means that the Cu(II)-imprinted plasmonic sensor can be detected by Cu(II) ions with a 96% accuracy in the range of 0.04–5 µM.

The limit of detection (LOD) and limit of quantification (LOQ) values were calculated as 3 and 10 s/b where s is the standard deviation of the plasmonic sensor response and can be measured as the y-intercept of the regression equation, which were obtained as 0.027 and 0.089 µM, respectively.

Furthermore, the coefficients were figured out for association and equilibrium (Scatchard) kinetic analysis and adsorption isotherm models (Langmuir and Freundlich) of the Cu(II)-imprinted plasmonic sensor. Association kinetic analysis depends on pseudo-first-order adsorption kinetics [36]. Equilibrium (Scatchard) kinetic analysis uses the experimental data for freely reversible host and guest binding interactions and calculates the total number of binding sites the host has in an equilibrium situation [37]. Langmuir adsorption isotherm model is based on the acceptation of homogeneous distribution of interaction points with equal energy and no lateral interactions. The Freundlich adsorption isotherm model is well fitted to heterogeneous surfaces [38]. Therefore, these calculations determine several parameters, including detection capability, surface homogeneity, and selectivity.

All calculations showed that the experimental data were coherent with the Langmuir adsorption isotherm model with the highest correlation coefficient (R^2^ = 0.9925). Moreover, the experimental ΔR_max_ (0.53) is close to the calculated ΔR_max_ (0.37) in the Langmuir model. These results showed that the binding properties of Cu(II) on plasmonic sensor surfaces are homogeneously distributed, monolayered, equal energy, and have minimal lateral interaction [39]. All adsorption isotherm models were demonstrated in Figures S4 and S5, respectively. The kinetic parameters for the Cu(II)-imprinted plasmonic sensor were also provided in Table S1. According to these results, it was seen that the theoretical ΔR_max_ value (0.55) calculated in the equilibrium kinetic analysis (Scatchard) is quite close to the experimentally obtained ΔR_max_ value (0.53). Likewise, according to the association kinetic analysis result, it was observed that the Cu(II) ion binds to the Cu(II)-imprinted plasmonic sensor surface with an accuracy of 97%.

**Figure 4:**
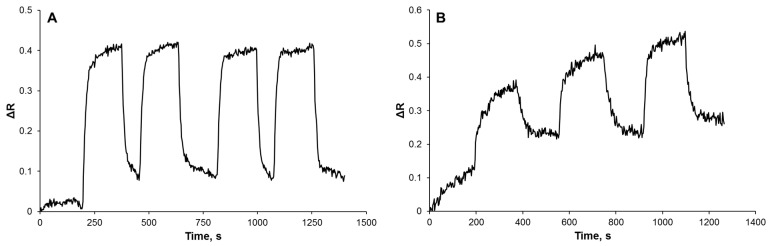
Reusability performance of Cu(II)-imprinted plasmonic sensor in the same (**A**) and different (**B**) Cu(II) concentrations.

To investigate the reusability of Cu(II)-imprinted plasmonic sensor, two separate analyses were carried out. Initially, the sensor surface reached equilibrium for 180 s, the Cu(II) sample was attracted to the plasmonic sensor for 180 s, and then the desorption process was carried out passing 0.01 mM EDTA through the system for 180 s. These processes were repeated four times in succession with the same concentration (4 µM). As can be seen in Figure 4A, the plasmonic sensor surface can be used repeatedly without any deterioration in the binding sites and, therefore, without any loss of performance. In the second experiment, the Cu(II) samples with increasing concentrations of 3, 4, and 5 µM were used, respectively, and it was observed that the ΔR changed incrementally and Cu(II) ion could also be determined without any performance loss (Figure 4B).

To show the storage stability, the Cu(II)-imprinted plasmonic sensor was tested with the same Cu(II) concentration (4 µM) at different times (0, 3, 24, and 36 months). The response of the Cu(II)-imprinted plasmonic sensor was decreased from only 0.42 to 0.26 over the past 36 months, and the performance loss was only 15%. According to this result, the Cu(II)-imprinted plasmonic sensor can be used for a long time for real-time Cu(II) ion detection with a tolerable performance loss (Figure S6).

**Figure 5:**
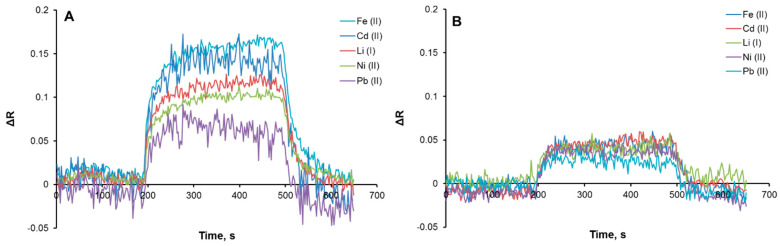
Selectivity performance of Cu(II)-imprinted (**A**) and non-imprinted (**B**) plasmonic sensors.

**Figure 6:**
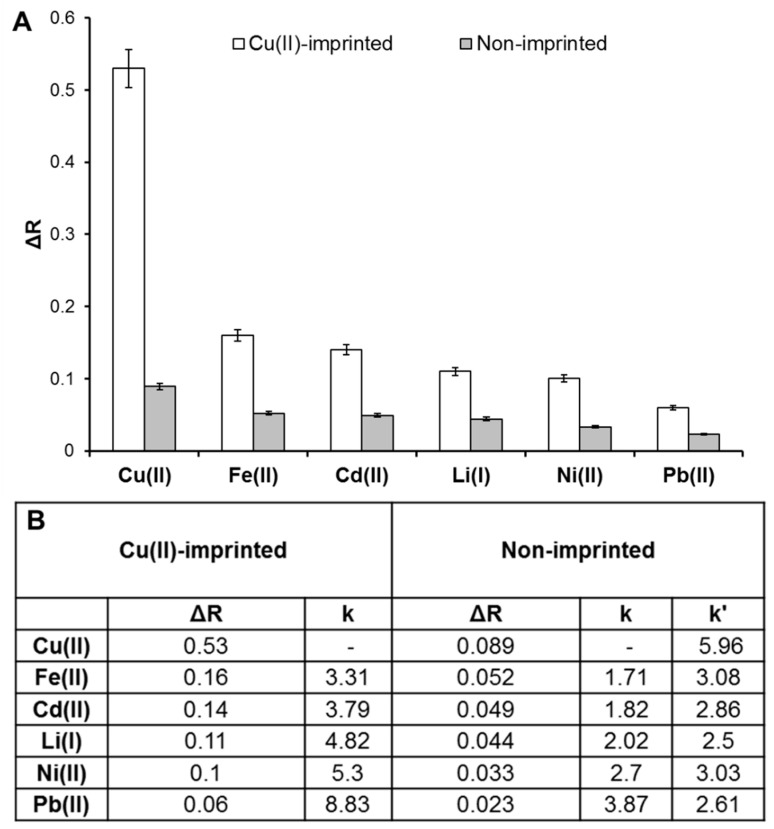
Selectivity performances (**A**) and selectivity coefficients (**B**) of Cu(II)-imprinted and non-imprinted plasmonic sensors.

For selectivity experiments, the non-imprinted plasmonic sensor was first interacted with a Cu(II) sample solution (5 μM) in pH 8.0 phosphate buffer, and it was observed that the ΔR decreased from 0.53 to 0.089, which means that the non-imprinted plasmonic sensor did not interact significantly with the Cu(II) ion with the resulting decrease in response (Figure S7). Afterward, competitive agents, including Fe(II), Cd(II), Li(I), Ni(II), and Pb(II), were also prepared at the same concentration (5 μM) and interacted with Cu(II)-imprinted (Figure 5A) and non-imprinted (Figure 5B) plasmonic sensors to obtain selectivity coefficients. According to the selectivity coefficient calculations (Figure 6), the Cu(II)-imprinted plasmonic sensor was more selective for Cu(II) than the other ions. These results showed that the chemical and physical feature of the Cu(II) was created on the plasmonic sensor surface.

**Figure 7:**
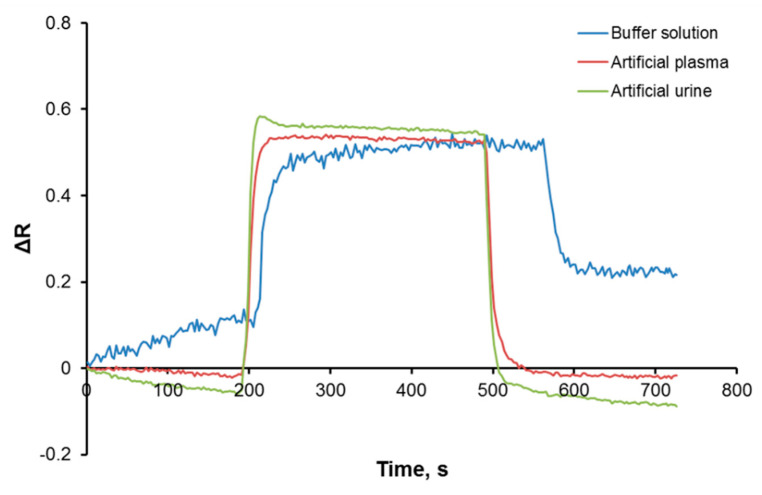
Real-time Cu(II) detection with Cu(II)-imprinted plasmonic sensor in complex media.

Real-time Cu(II) detection was also performed from artificial plasma and artificial urine samples with the Cu(II)-imprinted plasmonic sensor. According to the results, the Cu(II)-imprinted plasmonic sensor can detect Cu(II) not only in a buffer solution but also in complex media, including artificial plasma and/or urine (Figure 7). It was observed that other molecules in the same environment did not deform the binding sites of the Cu(II)-imprinted plasmonic sensor, and there was no loss in the detection performance.

## 4. Conclusions

Metals are universal in humans and nature. Cu(II), the third most abundant trace metal element in the human body, is essential to human health but an excess Cu(II) concentration can cause damage to the organ. From the environmental perspective, Cu(II) can also depress the self-purification ability of natural waters. Thus, it is a vital step to prepare a sensitive and selective sensor for Cu(II) detection. In recent years, many advanced sensors have been successfully designed for Cu(II) detection. In this present study, an ion-imprinted polymer integrated plasmonic sensor was prepared using an amino acid-based functional monomer for real-time and fast Cu(II) detection. Several characterization experiments showed that the molecularly imprinted polymer was successfully and homogeneously synthesized on the gold surfaces of the plasmonic sensor. Furthermore, the well-designed Cu(II)-imprinted plasmonic sensor displayed a wide detection range from 0.04 to 5.0 μM with a low detection limit of 0.027 μM with a short response time (20 s). This study has not only validated the analytical principle but also proposed a convenient and selective alternative to detecting Cu(II) in artificial samples.

Finally, a comparison table was prepared with several parameters, including materials, detection range, limit of detection, selectivity, and real samples, to examine optic sensors (Table 1). Since this study focuses on the ion-imprinted plasmonic sensor, these parameters were assessed with the other sensors having closer properties. Different optic sensors can be prepared using various materials, such as nanoparticles and quantum dots, to detect Cu(II) ions in several media. It was seen that there are a limited number of studies on Cu(II) detection with imprinted polymers. When compared with other optic sensors, it was concluded that the Cu(II)-imprinted plasmonic sensor can go down to lower detection limits with a wider range than those used in other studies. The use of imprinted polymers made the sensor more selective and sensitive in the detection.

## Figures and Tables

**Table 1:** Comparison of optic sensors for Cu(II) detection.

Ref.	Material	Detection Range	Limit of Detection	Selectivity	Real Sample
[40]	Silicon nanoparticles	0.1–200 µM	0.1 μM	Fe^2+^, Na^+^, K^+^, Mg^2+^, Mn^2+^, Ca^2+^	Tap water
[41]	Graphene quantum dot	0–0.20 mM	0.33 μM	Cr^+3^, Ba^+2^, Ca^+2^, Cd^+2^, Co^+2^, K^+^, Mn^+2^,Ni^+2^, Pb^+2^, Zn^+2^, Fe^+3^, Ag^+^, Hg^+2^	River water
[42]	Silver nanoparticles	0.08–1.44 μM	0.16 μM	Mn^+2^, Mo^+3^, Na^+^, Cr^+3^, Hg^+2^, Ni^+2^, Ca^+2^, K^+^, Cs^+^, Li^+^, As^+^, PO_4_^−3^, NH_4_, NO_3_^-^	Tap and pond water
[43]	CdS quantum dot	1–100 mg/L	-	Zn^+2^, Mn^+2^, Ni^+2^, Fe^+2^, Fe^+3^, I, Pb^+2^, Al^+3^, Mg^+2^,Ca^+2^, K^+^, Na^+^	Potatoes
[44]	Graphene oxide	0–1.18 μM	54 nM	Na^+^, K^+^, Ca^+2^, Mn^+2^,Co^+2^,Fe^+2^, Fe^+3^, Zn^+2^, Al^+3^, Cr^+6^,As^+5^, Cd^+2^, Zn^+2^, Pb^+2^, Hg^+2^	Water
This study	Imprinted polymer	0.04-5 μM	0.027 μM	Fe^+2^,Cd^+2^, Li^+1^, Ni^+2^, Pb^+2^	Artificial plasma and urine

## Data Availability

Not applicable.

## Supplementary Materials

The following supporting information can be downloaded at: https://www.mdpi.com/article/10.3390/bios12020091/s1, Figure S1: Ultraviolet-visible spectroscopy result of MAC-Cu(II) pre-complex; Figure S2: ATR-FTIR spectra of MAC monomer, Cu(II)-imprinted and non-imprinted plasmonic sensors; Figure S3: Sensorgrams (A) and bar graphs (B) of Cu(II)-imprinted plasmonic sensor in real-time Cu(II) detection at different pHs; Figure S4: Equilibrium (Scatchard) (A) and association kinetic (B) analysis of Cu(II)-imprinted plasmonic sensor; Figure S5: Langmuir (A) and Freundlich (B) adsorption isotherm models of Cu(II)-imprinted plasmonic sensor; Figure S6: Storage stability of Cu(II)-imprinted plasmonic sensor; Figure S7: Real-time Cu(II) detection by Cu(II)-imprinted and non-imprinted plasmonic sensors. Table S1: The kinetic parameters of Cu(II)-imprinted plasmonic sensor.
