# Supplementary material for: Ion-Imprinted Polymer-on-a-Sensor for Copper Detection
Source: Biosensors (Basel). 2022 Feb 2;12(2):91. doi: 10.3390/bios12020091 (PMC8869677; doi:10.3390/bios12020091)
Supplement: Supplementary file 1 [file biosensors-12-00091-s001.zip › biosensors-1567288-supplementary.pdf]

Ion-Imprinted Polymer-on-a-Sensor for Copper Detection

Zeynep Gerdan[1], Yeşeren Saylan[2], Mukden Uğur[3], Adil Denizli[2,*]

Supplementary file

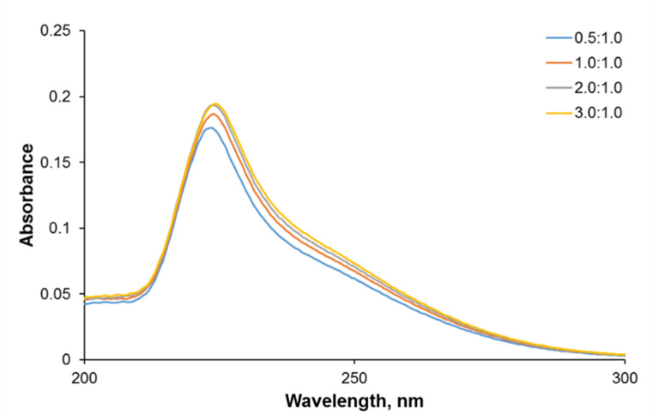

Figure S1. Ultraviolet-visible spectroscopy result of MAC-Cu(II) pre-complex.

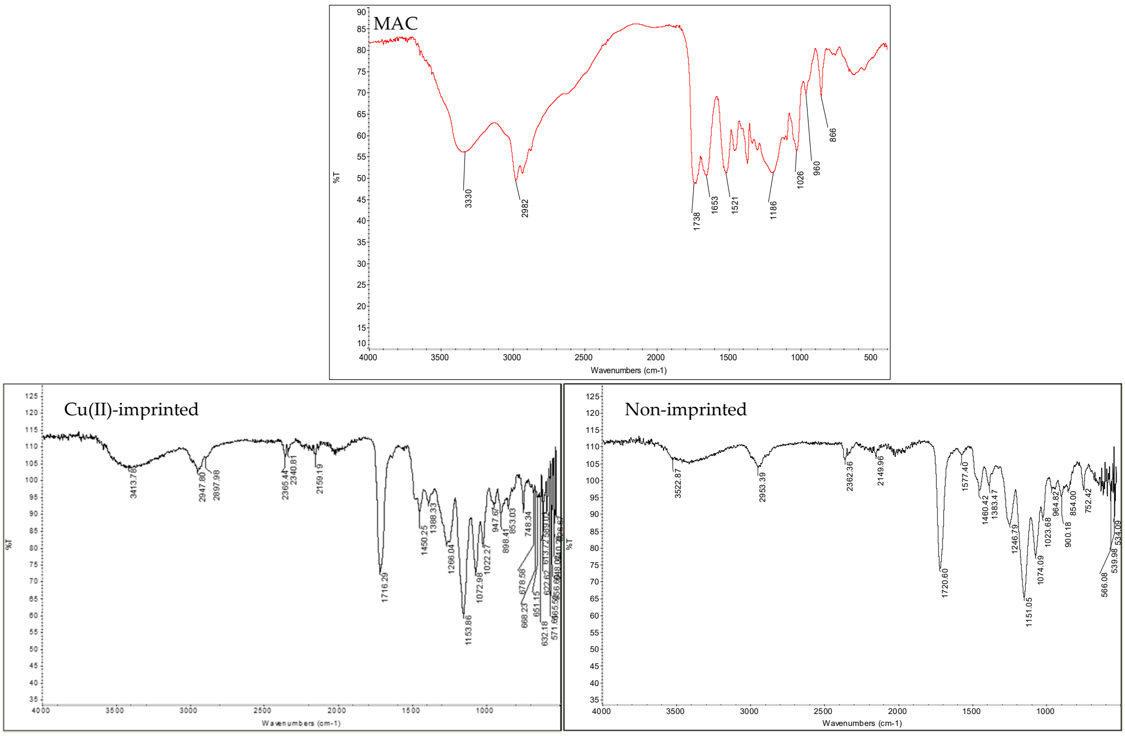

Figure S2. ATR-FTIR spectra of MAC monomer, Cu(II)-imprinted and non-imprinted plasmonic sensors.

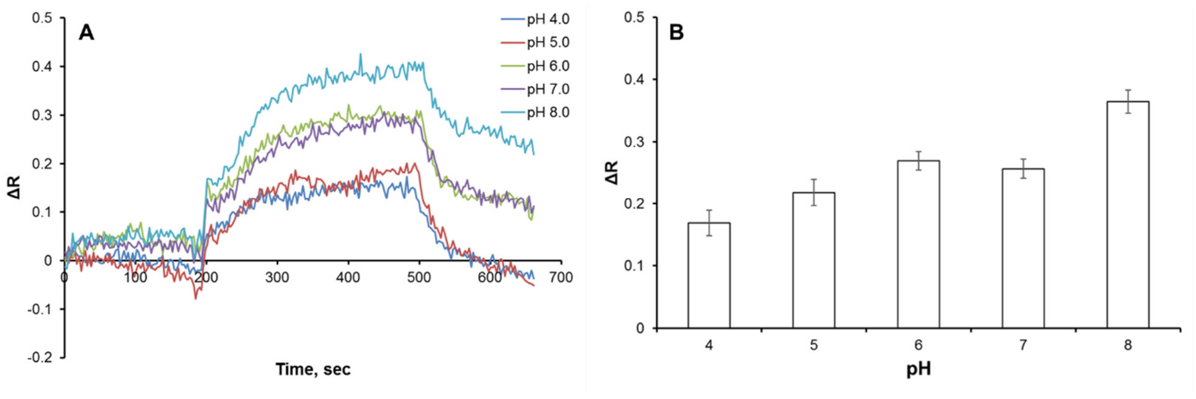

Figure S3. Sensorgrams (A) and bar graphs (B) of Cu(II)-imprinted plasmonic sensor in real-time Cu(II) detection at different pHs.

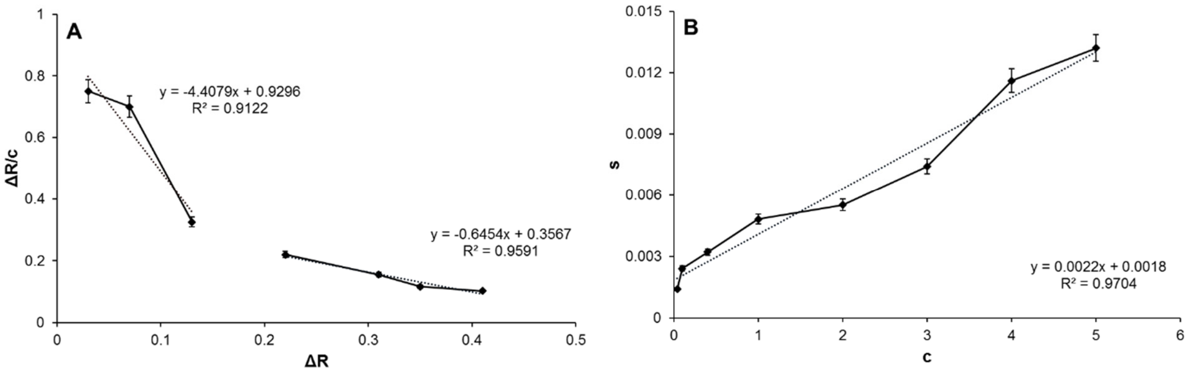

Figure S4. Equilibrium (Scatchard) (A) and association kinetic (B) analysis of Cu(II)-imprinted plasmonic sensor.

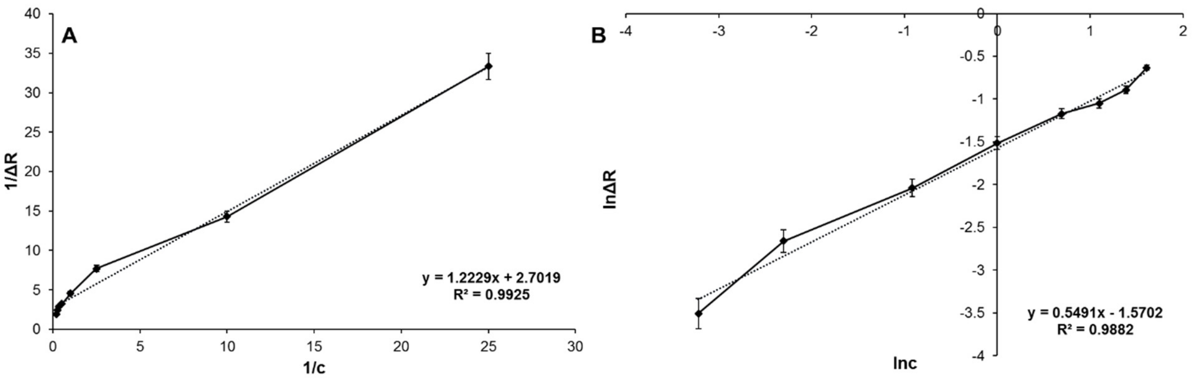

Figure S5. Langmuir (A) and Freundlich (B) adsorption isotherm models of Cu(II)-imprinted plasmonic sensor.

Table S1. The kinetic parameters of Cu(II)-imprinted plasmonic sensor.

Scatchard		Association		Langmuir		Freundlich	
ΔR_{max}	0.55	k_a, μM^{-1}. s^{-1}	0.002	ΔR_{max}	0.37	ΔR_{max}	4.81
K_A, μM^{-1}	0.65	k_d, s^{-1}	0.002	K_D, μM	0.45	$1/n$	0.55
K_D, μM	1.55	K_A, μM^{-1}	1.22	K_A, μM^{-1}	2.21	R^2	0.98
R^2	0.96	K_D, μM	0.82	R^2	0.99		
		R^2	0.97				

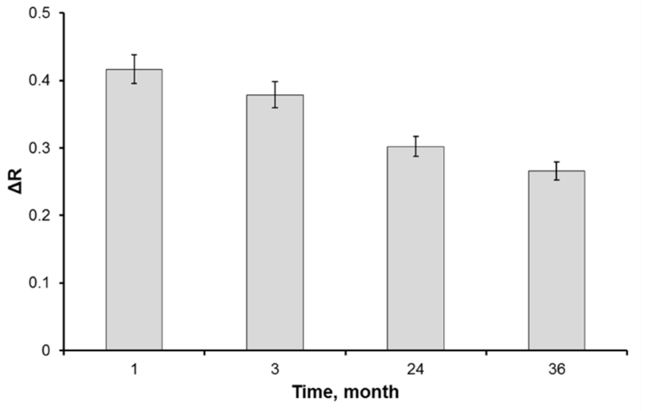

Figure S6. Storage stability of Cu(II)-imprinted plasmonic sensor.

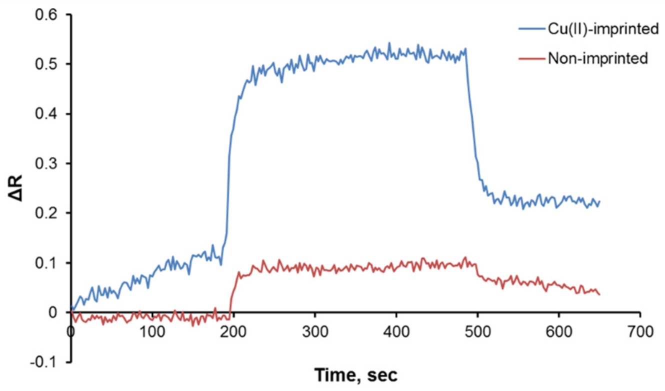

Figure S7. Real-time Cu(II) detection by Cu(II)-imprinted and non-imprinted plasmonic sensors.
